# Complete genome analysis reveals evolutionary history and temporal dynamics of Marek’s disease virus

**DOI:** 10.3389/fmicb.2022.1046832

**Published:** 2022-11-03

**Authors:** Kai Li, Zhenghao Yu, Xingge Lan, Yanan Wang, Xiaole Qi, Hongyu Cui, Li Gao, Xiaomei Wang, Yanping Zhang, Yulong Gao, Changjun Liu

**Affiliations:** Avian Immunosuppressive Diseases Division, State Key Laboratory of Veterinary Biotechnology, Harbin Veterinary Research Institute, Chinese Academy of Agricultural Sciences, Harbin, China

**Keywords:** Marek’s disease virus, genomic analysis, evolution, recombination, temporal dynamics

## Abstract

Marek’s disease has caused enormous losses in poultry production worldwide. However, the evolutionary process and molecular mechanisms underlying Marek’s disease virus (MDV) remain largely unknown. Using complete genomic sequences spanning an unprecedented diversity of MDVs, we explored the evolutionary history and major patterns in viruses sampled from 1964 to 2018. We found that the evolution of MDV strains had obvious geographical features, with the Eurasian and North American strains having independent evolutionary paths, especially for Asian strains. The evolution of MDVs generally followed a clock-like structure with a relatively high evolutionary rate. Asian strains had evolved at a faster rate than European strains, with most genetic mutations occurring in Asian strains. Our results showed that all recombination events occurred in the UL and US subregions. We found direct evidence of a closer correlation between Eurasian strains, related to a series of reorganization events represented by the European strain ATE2539. We also discovered that the vaccine strains had recombined with the wild virulent strains. Base substitution and recombination were found to be the two main mechanisms of MDV evolution. Our study offers novel insights into the evolution of MDVs that could facilitate predicting the spread of infections, and hence their control.

## Introduction

Herpesviruses have evolved over 400 million years, with alphaherpesviruses, an extensive subfamily containing numerous mammalian and avian viruses, reported to have been separated from the other branches 180–210 million years ago (McGeoch et al., 1995, 2000; Weir, 1998). Marek’s disease virus (MDV), also known as *Gallid alphaherpesvirus 2*, is a highly pathogenic and oncogenic herpesvirus that causes Marek’s disease (MD), a highly contagious malignant T-cell lymphomatosis in chickens (Nair, 2018). The MDV genome has been shown to be a linear, double-stranded DNA molecule with a class E architecture, divided into 6 regions, including the terminal repeat long (TRL), unique long (UL), internal repeat long (IRL), internal repeat short (IRS), unique short (US), and terminal repeat short (TRS) regions (Lole et al., 1999; McGeoch et al., 2006; Davison, 2010).

Marek’s disease virus remains a threat to the poultry industry due to its evolution toward higher virulence. Based on their pathogenicity, MDV viral strains are currently classified into four pathotypes ranging from mild (m), virulent (v), very virulent (vv), to very virulent plus (vv+) (Witter, 1997). Vaccination has been shown to generate an anti-tumor immune response following infection with field strains, which reduces mortality rates; however, it does not protect against infection, resulting in the evolution of viruses with increased replication and virulence (Gimeno, 2008). Both vaccination and intensive farming could dramatically influence pathogen transmission because they artificially manipulate the immune status and population dynamics of the host. The impact of these factors on transmission could, in principle, result in an increase in pathogen virulence.

The mechanisms responsible for an increase in MDV virulence and concomitant changes in disease have attracted the increased interest of researchers over the last few decades. The virulence of MDV has been suggested to be a polygenic trait, with allelic variants at several loci playing a role (Shamblin et al., 2004; Liao et al., 2021). Homologous recombination has also been suspected to play an important role in the evolution of virulence, because it can bring together new mutations that originally evolved against different genetic backgrounds. Thus, epidemiological surveys of MDV are deemed necessary. However, the available genetic data, especially for emerging strains of MDV with apparent different pathogenic characteristics and increased virulence remain insufficient.

Large-scale analysis of MDV, particularly comprising complete genome sequences, is needed to reveal the origins, processes, and patterns of its evolution. The objectives of this study were to expand the database of MDV genomic sequences using complete DNA sequencing of MDV isolates and determine whether MDV sequences varied among isolates from different geographic locations or over time. We presented the evolutionary studies of the complete genomes of MDV, utilizing a data set of 58 whole-genome sequences sampled from three continents over a time period of 54 years. The size of the data set allowed us to reveal the evolutionary rates among MDV and determine the complex evolutionary dynamics of MDV.

## Materials and methods

### Ethical statement

Specific pathogen free (SPF) duck eggs were purchased from the National Laboratory Poultry Animal Resource Center (Harbin, China). Duck embryo fibroblasts (DEFs) were prepared from 10-days-old specific pathogen-free (SPF) duck embryos (Shahsavandi et al., 2013). This study was carried out following the recommendations in the Guide for the Care and Use of Laboratory Animals of the Ministry of Science and Technology of China. The use of SPF duck eggs was approved by the Animal Ethics Committee of Harbin Veterinary Research Institute of the Chinese Academy of Agricultural Sciences and was performed in accordance with animal ethics guidelines and approved protocols [SYXK (Hei) 2017-009].

### Samples

Marek’s disease virus strains were propagated on DEFs in M199 containing 2% fetal bovine serum (FBS, Sigma-Aldrich, St. Louis, MO, USA). The complete genome sequences of a total of 58 MDV isolates collected from three continents between 1964 and 2018 were analyzed. Among these MDV isolates, 26 strains were isolated from Chinese poultry farms during 2006 to 2018 and sequenced in the present study.

### DNA extraction and next-generation sequencing

Viral DNA was isolated from the infected DEFs using a micrococcal nuclease procedure (Volkening and Spatz, 2009). Briefly, after permeabilization, cellular DNA was degraded using micrococcal nuclease. The viral capsid was digested by proteinase K to release viral DNA, and high molecular weight viral DNA was precipitated using PEG-8000. Sequencing of the viral DNA was carried out using the Illumina HiSeq2000 sequencing platform (San Diego, CA, USA). The assembly results were optimized according to paired-end and overlap relationships by mapping the reads to contigs after removing the host sequence. Repetitive regions and ambiguities in the genome were re-sequenced using traditional Sanger sequencing.

### Genome sequence alignment and phylogenetic analysis

Alignment of genome sequences was performed using MAFFT (Kumar et al., 2008). For phylogenetic analysis, alignment gaps associated with incomplete genomic data, variable repeat regions, mini-F vector sequences, and reticuloendotheliosis virus (REV) long terminal repeats were removed using Gblocks v0.91 (Castresana, 2000). An initial phylogenetic tree of these sequences was inferred using the maximum likelihood procedure available in the PhyML package (Guindon et al., 2010). This analysis used the HKY + Γ4 model of nucleotide substitution and NNI + SPR branch-swapping. Using a regression of root-to-tip genetic distances on the ML tree in TempEst (Rambaut et al., 2016), the degree of clock-like structure in the data was inferred above against the year of virus sampling. Under this analysis, the correlation coefficient indicated the amount of variation in genetic distance, which is explained by sampling time. The rates and dates of viral evolution were analyzed using the Bayesian Markov chain Monte Carlo (MCMC) approach available in the BEAST package (Drummond et al., 2012). For this analysis, a range of nucleotide substitution (HKY + Γ4, GTR + Γ4), molecular clock (strict, relaxed uncorrelated lognormal), and demographic (constant, Bayesian skyride) models were used. All analyses were run twice and for sufficient time (100 million generations) to ensure that convergence was achieved, with statistical uncertainty manifesting in values of the 95% highest posterior distribution (HPD). Ancestral pathotypes and geographical origins were inferred in BEAST by reconstructing discrete traits onto the final rooted time-measured MDV phylogeny using a symmetric substitution model (Lemey et al., 2009). MCMC was run and combined as described above, followed by mapping of inferred ancestral traits onto a maximum clade credibility (MCC) tree. Geographical locations and pathotypes based on a standardized *in vivo* virulence grading scheme were inferred from the literature, similar to pathotypes, based on a standardized *in vivo* virulence grading scheme (Witter et al., 2005; Nair, 2018). Phylogenetic analysis of alignments of single gene and SNP tandem sequences was conducted using the neighbor-joining method in MEGA6 (Kumar et al., 2008). All phylogenetic reconstructions were statistically assessed through the analysis of 1,000 bootstrap replications.

### Recombination analysis

To accurately identify potential recombinants, the sequences of the 26 strains identified in this study were combined with up to 32 isolate sequences for recombination screening. Recombination networks on alignments of complete genomes, and the sequences in different sub-regions (UL, IRL, IRS, and US) of the 58 MDV strains were performed using SplitsTree 4 (Huson, 1998). Statistical analysis of the recombination networks was performed using the Phi test. Putative recombination events were identified using the Recombination Detection Program (RDP). To further analyse the possibility of recombination, bootscan analysis was performed to detect the crossover points for recombination events of selected sequences as representatives of different clusters using Simplot (Lole et al., 1999).

## Results

### Genome organization

We found that the complete nucleotide sequences of the 58 MDV strains were similar in size and organized into six regions. As the TRL and TRS regions are known to be identical to the IRL and IRS regions, respectively, these regions were not used in subsequent analysis. Details of the 58 strains and their GenBank accession numbers are presented in Supplementary Table 1. Among these viral strains, 26 were newly sequenced in this study and had been isolated in a time span of 12 years from 2006 to 2018, with each year having a representative strain. The evolution of these strains was continuously monitored in real time.

### Evolutionary dynamics of Marek’s disease virus

**FIGURE 1:**
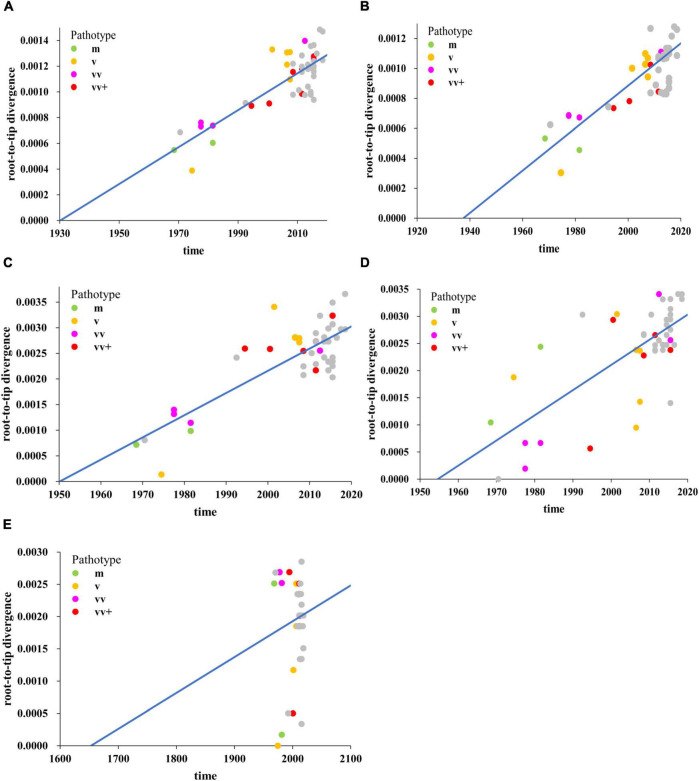
Regression of root-to-tip genetic distances against year of sampling for Marek’s disease virus (MDV) strains as inferred from the ML phylogenetic tree, including complete genome **(A)**, unique long (UL) region **(B)**, internal repeat long (IRL) region **(C)**, internal repeat short (IRS) region **(D)**, and unique short (US) region **(E)** sequences. The mild (m, green), virulent (v, yellow), very virulent (vv, pink), very virulent plus (vv+, red) MDV, and 26 MDV strains isolated and sequenced in this study (gray) were analyzed.

To better understand the relationship among the individual MDV strains isolated from different periods of time and different regions (Asia, Europe, and North America), as well as the evolutionary dynamics of MDV, we performed sequence alignments of the complete genome and different subregional (UL, IRL, IRS, and US) genomic sequences. We first determined whether our obtained datasets contained sufficient information on temporal structure to undertake detailed molecular clock analyses by performing a regression of root-to-tip genetic distance against the year of sampling. We found that the temporal signal generated by the complete genome datasets was very high, with a correlation coefficient of 0.83 (Figure 1A). Analysis of subregions revealed that the temporal signals of the UL (Figure 1B; correlation coefficient = 0.77), IRL (Figure 1C; correlation coefficient = 0.81), and IRS (Figure 1D; correlation coefficient = 0.72) subregions were also high; however, the temporal signal of the US subregion was shown to be very low (Figure 1E; correlation coefficient = 0.11). These data indicated that a time-scaled phylogenetic analysis was appropriate for the complete genome, the UL, IRL, and IRS subregions, but not for the US subregion. For dated phylogenetic reconstructions in BEAST, we used a GTR + Γ4 site model, a constant size coalescent tree prior, and relaxed uncorrelated lognormal clock (Drummond et al., 2006). These were selected following Bayes factor comparison of marginal-likelihood estimates. We estimated the mean evolutionary rate of complete genomes for the selected evolutionary model to be 8.25 × 10^–6^ substitutions per site per year with 95% HPD values between 3.78 × 10^–6^ and 1.56 × 10^–5^ (Supplementary Table 2).

### Phylogenetic analyses of Marek’s disease virus

**FIGURE 2:**
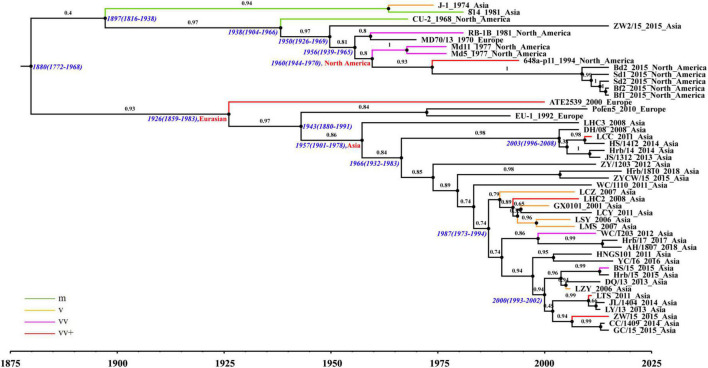
Maximum clade credibility (MCC) tree of Marek’s disease virus (MDV) strains based on complete genomic sequences using the evolutionary model GTR + Γ4.

The phylogenetic analysis based on complete genomes divided the strains into two major clades (Figure 2). The results obtained from the maximum clade credibility (MCC) tree showed that the evolution of MDV had obvious geographical features. At the complete genomic level, Eurasian strains were demonstrated to be closely related, having evolved in different directions from those of North American strains. Clade I consisted of all North American strains except for two early Asian strains (J-1 and 814), one exceptional Asian strain (ZW2/15), and one early European strain (MD70/13). All MDV strains in clade II were found to be Eurasian strains with European strains being clustered in the European subclade, and all Asian strains clustered in the Asian subclade. Notably, the European strain ATE2539 located in a separate subclade with no other strains, indicating that the genome sequence of this strain could be different from other Eurasian strains. The phylogenetic trees established based on the sequences of the UL subregion were generally similar to those based on the complete genome (Supplementary Figure 1A). Accordingly, we found that the clustering of strains in clade I and clade II were consistent with the results of the complete genome evolutionary tree, further confirming the close relationship between the Asian and European strains. The results of the IRS phylogenetic tree were also divided into two clades (Supplementary Figure 1B). All Eurasian strains, except LSY and MD70/13, located in clade II, whereas the remaining strains isolated in North America located in clade I. Although the IRL subregion phylogenetic tree differed from that based on the complete genome regarding large clades, it could be clearly seen that the Eurasian strains had a different evolutionary path from that of the North American strains (Supplementary Figure 1C).

The North American and Eurasian strains had independent evolutionary paths (Figure 2); this was especially apparent for the Asian prevalent strains after 2000. Ancestral reconstruction of roots traced back their origin to the Eurasian continent, with the Asian strains shown to have been derived from European strains. Based on the estimation of a reliable substitution rate, we determined the mean times to common ancestry (tMRCA) for each MDV clade and subclade. The tMRCA of the MDV strains analyzed in this study was estimated to be 1880 with 95% HPD between 1772 and 1968, which is 27 years before the first report of MDV (Marek, 1907; Biggs, 2001). The tMRCA of the Eurasian clade was identified to be 1926, with 95% HPD between 1859 and 1983. The tMRCA of the Asia subclade was estimated to be 1957 with 95% HPD between 1901 and 1978, which is about 10 years earlier than the full introduction of vaccines in Asia and a decade later than the earliest records of increased disease severity in reared poultry, as in Europe and North America.

**FIGURE 3:**
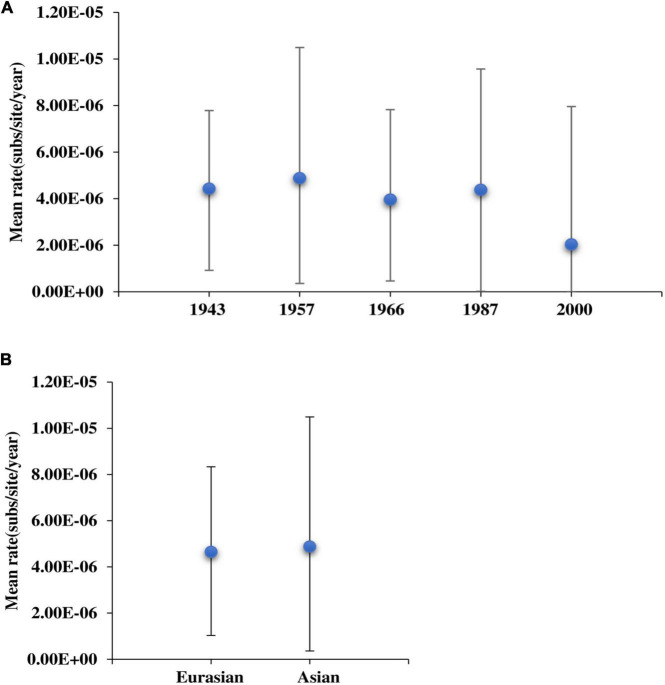
The evolutionary rate of Marek’s disease virus (MDV) strains. **(A)** Analyses of the evolutionary rates of MDV strains. **(B)** Comparison of the evolutionary rate between Eurasian and Asian strains.

To explore the relationship between virulence enhancement and evolutionary rate, we estimated the evolutionary rate of MDV strains and found that it fluctuated regularly (Figure 3A). Based on the pathogenic evolutionary pattern (Nair, 2005), we observed that the volatility of the evolutionary rate was roughly the same as the time regular of virulence enhancement. We found that at each virulence enhancement period, the corresponding evolutionary rate was fast, whereas the corresponding evolutionary rate of the plateau was relatively low. Moreover, the rate of evolution of Asian branches was shown to be higher than that of Eurasian strains (Figure 3B), indicating that Asian strains had evolved faster than European strains.

### Mutation analyses of Marek’s disease virus

**FIGURE 4:**
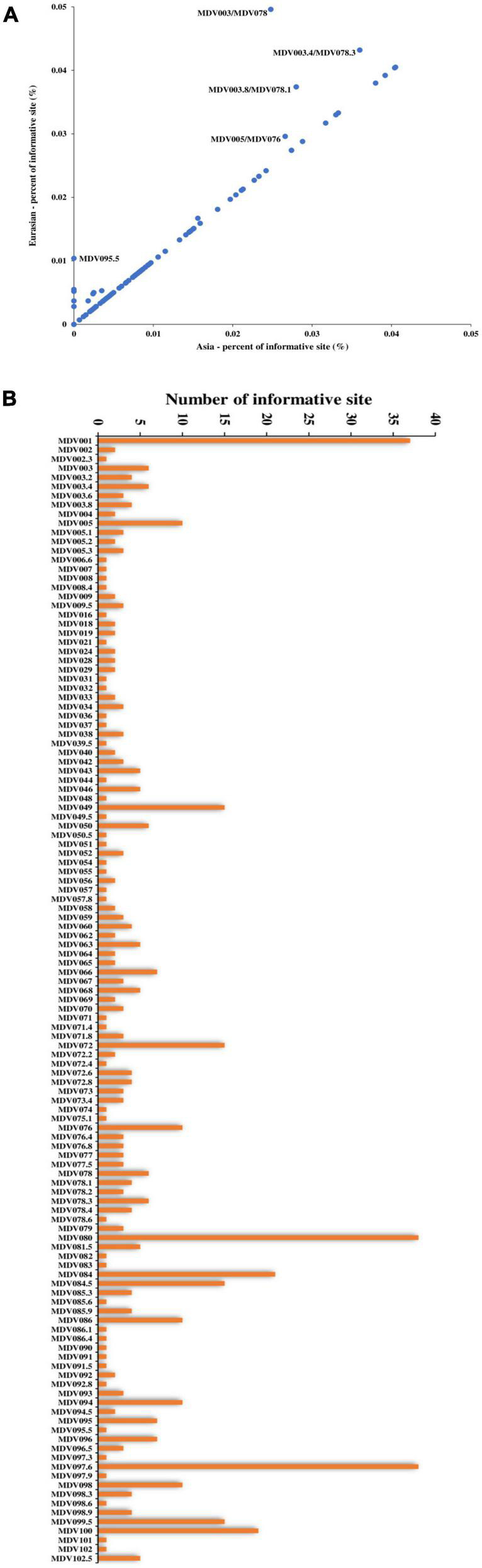
Single nucleotide polymorphism analyses of Marek’s disease virus (MDV) strains. **(A)** Comparison of point mutations between Eurasian and Asian strains. **(B)** List of MDV ORFs with mutation sites.

We performed single nucleotide polymorphism (SNP) analysis on the open reading frames (ORFs) of all strains and then employed phylogenetic tree analysis. Our results showed a similar geographical evolution with that obtained in complete genome analysis (Supplementary Figure 2). A comparison of point mutations between Eurasian and Asian strains revealed that most genetic mutations occurred in Asian strains, as most genes were located on a 45-degree diagonal (Figure 4A). To determine whether the variation in the rates of evolutionary change might have resulted from differing selection pressures, we compared the ratio of non-synonymous (dN) to synonymous (dS) substitutions between Eurasian and Asian strains. We found that most of the genes with a relatively large number of mutation sites, such as MDV003.4/078.3 (RLORF4), MDV005/076 (Meq), and MDV084/100 (ICP4), located in repetitive regions. However, we also identified some genes located in the unique region, such as MDV049 (UL36), MDV066 (UL52), and MDV094 (gD) that were characterized by many mutation points (Figure 4B). Phylogenetic analysis of MDV084/100 (ICP4), MDV072 (LORF11), MDV049 (UL36), and MDV094 (gD) showed that the ZW2/15 strain was closely related to the early Asian strain J-1, vaccine strain 814, and North American strains, consistent with the results of complete genome analysis (Supplementary Figure 3).

### Recombination analysis of Marek’s disease virus

**FIGURE 5:**
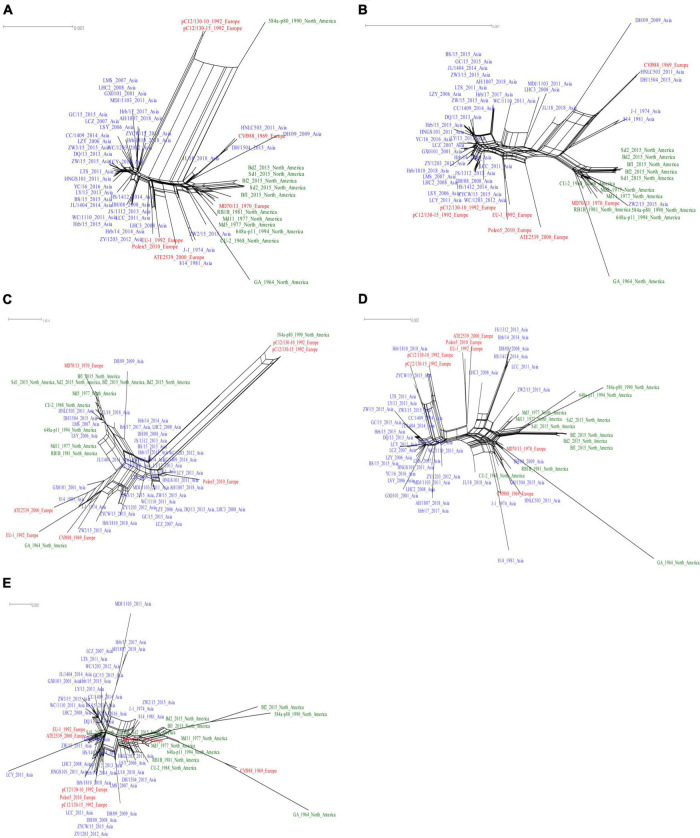
Recombination network analyses of Marek’s disease virus (MDV) strains. Results are shown based on complete genome **(A)**, unique long (UL) **(B)**, unique short (US) **(C)**, internal repeat long (IRL) **(D)**, and internal repeat short (IRS) **(E)** sub-regions.

We next analyzed the recombination network of the complete genome and different genomic subregions of MDV strains. We identified a total of 1,075 informative sites in the complete genomes of the 58 MDV strains, with the performed phi test presenting statistically significant evidence for recombination (p < 10^–5^; Figure 5A). We also detected the occurrence of recombination events in both UL (396 informative sites, p < 10^–5^; Figure 5B) and US (315 informative sites, p < 10^–5^; Figure 5C) regions. Although we detected the presence of 128 informative sites in the IRL region (Figure 5D) and 220 informative sites in the IRS region (Figure 5E), the evidence obtained for the occurrence of recombination of these two subregions was not significant (p > 10^–5^). The topologies of phylogenetic networks in the complete genome and in different sub-regions of all MDV strains generated using the Splits Tree were shown to be consistent with the relationships analyzed using MAFFT.

**FIGURE 6:**
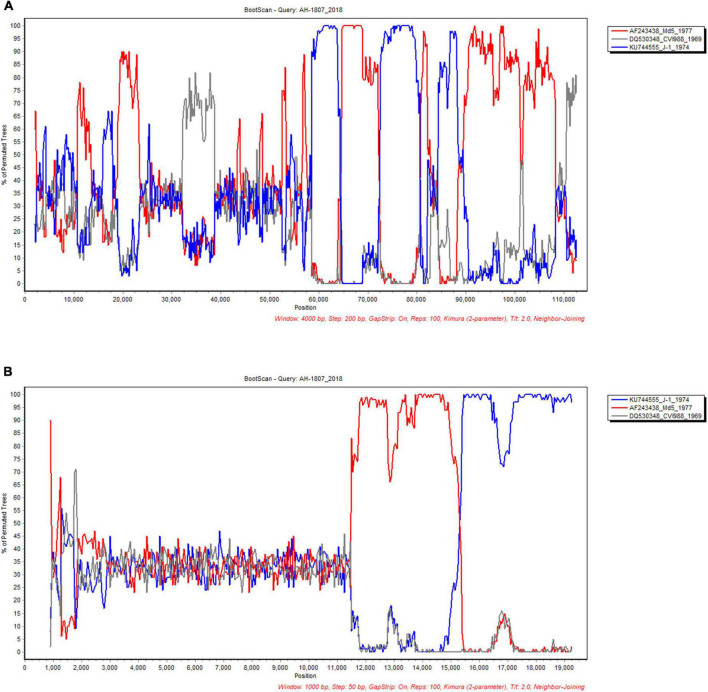
Analyses of recombination crossover points in the unique long (UL) and unique short (US) sub-regions. The UL **(A)** and US **(B)** sub-region of AH/1807 strain were selected as query, and the corresponding sub-regions of the early Asian strain J-1, North American strain Md5, and vaccine strain CVI988 were chosen to perform bootscan analysis using SimPlot.

In order to define the recombination crossover points in the UL and US subregions, we selected the AH/1807 strain as the query, and chose the early Asian virulent strain J-1, North American virulent strain Md5, and European vaccine strain CVI988 to perform bootscan analysis using SimPlot. Our bootscan analysis revealed pairs of crossover points in the UL (Figure 6A) and US (Figure 6B) regions. Likewise, we selected the AH/1807 strain as a query, and chose the early European strain ATE2539, North American virulent strain GA, and European vaccine strain CVI988 to perform bootscan analysis using SimPlot. In this case, our bootscan analysis revealed pairs of crossover points in the UL (Supplementary Figure 4A) and US (Supplementary Figure 4B) regions, further confirming the occurrence of recombination between the UL and US regions.

**FIGURE 7:**
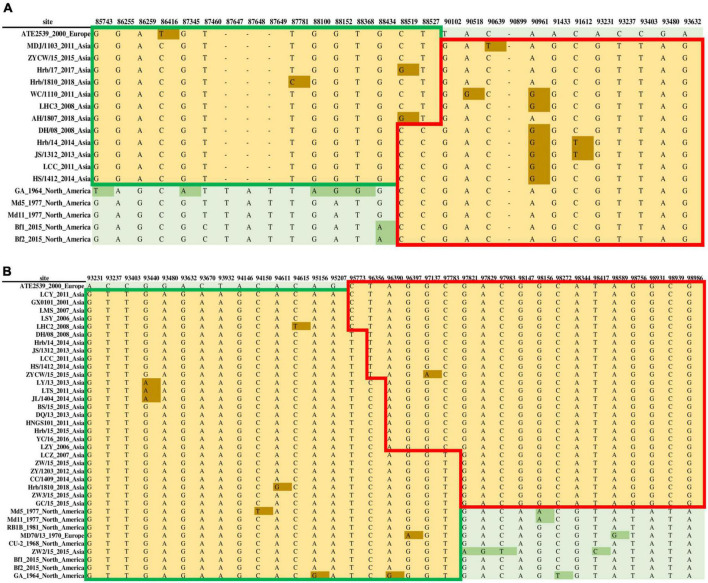
Two forms of recombination in the unique long (UL) sub-region involving the Marek’s disease virus (MDV) European strain ATE2539. **(A)** The recombination of the European strain ATE2539 with North American strains produced Asian strains. **(B)** The recombination of the European strain ATE2539 with North American strains, the European strain MD70/13, and Asian strain ZW2/15 produced the prevalent Asian strains.

To accurately identify all potential recombinants, we combined the genomic sequences of the 26 MDV strains identified in this study with 32 genomic sequences obtained from GenBank for recombination screening. We used the vaccine strain CVI988 as a reference strain and arranged them according to the UL and US sequences of each strain. We obtained a recombination model that was roughly divided into two cases: the first case regarded recombination events involving the European strain ATE2539, whereas the other regarded recombination events involving the early Asian virulent strain J-1 and Asian vaccine strain 814. We identified two forms of recombination in the UL sub-region involving ATE2539. The first was the recombination of the European strain ATE2539 and North American strains as parent strains to produce Asian strains, utilizing different recombination sites (Figure 7A). The other observed form was the recombination of the European strain ATE2539 with North American strains, the European strain MD70/13, and the Asian strain ZW2/15 to produce the most prevalent Asian strains, also utilizing different recombination sites (Figure 7B).

We detected three forms of recombination involving the UL subregion of the early Asian strain J-1 and vaccine strain 814. The first was the recombination of the early Asian virulent strain J-1 with North American, European (CVI988, ATE2539, EU-1, MD70/13, and Polen5), and Asian (DH/1504, HNLC503, and WC/1110) strains to produce some Asian and two European (pc12/130-10 and pc12/130-15) strains (Supplementary Figure 5A). The second form regarded the recombination of the early Asian virulent strain J-1 with North American and Asian strains (LHC3 and ZW2/15) to generate some Asian strains (Supplementary Figure 5B). The third was the recombination of the early Asian virulent strain J-1 and vaccine strain 814 with North American and European (MD70/13) strains to produce some Asian and two European (pC12/130-10 and pC12/130-15) strains (Supplementary Figure 5C).

Similarly, we detected the occurrence of two recombination forms involving the US region. The first form regarded the recombination between the European strain ATE2539 and North American strains to produce some Asian strains, utilizing different recombination sites (Supplementary Figure 6A). Whereas, the other form involved the recombination of the J-1 strain, 814 strain, early North American strain GA, Asian strain ZW2/15, European strain EU-1, North American strains, three Asian strains (HNLC503, DH/1504, and LSY), and two European strains (ATE2539 and MD70/13) to produce some Asian and three European (Polen5, pC12/130-10, and pC12/130-15) strains (Supplementary Figure 6B). The hotspots of recombination in the UL and US regions are shown in Table 1.

**TABLE 1:** The hot spots of recombination in the unique long (UL) and unique short (US) subregions of Marek’s disease virus (MDV) genome.

Subregion	ORF	Gene	Location
UL	MDV049	UL36	64508–74971
	MDV056	UL43	87240–88502
	MDV057	UL44	88723–90230
	MDV060	UL47	93710–96136
	MDV061	UL48	96375–97658
US	MDV094	gD	14162–15375
	MDV094.5	Hypothetical protein	16666–16956
	MDV095	gI	15482–16549
	MDV095.5	Hypothetical protein	16666–16956
	MDV096	gE	16688–18181
	MDV096.5	Hypothetical protein	17640–17915

The above results showed that there were seven recombination types associated with the MDV strains analyzed in the present study, five of which were recombination with the European strain ATE2539 as parent. We identified three recombination types specifically using the ATE2539 strain as parent; in these types the ATE2539 strain could not be replaced by other strains. To investigate the reasons behind the use of ATE2539 as a parent strain in recombination events to form prevalent strains in Asia, we conducted a recombination analysis of ATE2539 and found that ATE2539 was derived from the recombination in the UL subregion between early North American virulent strain GA and early Asian virulent strain J-1 (Supplementary Figure 7). This finding revealed that the European strain ATE2539 has an Asian ancestry, thus providing a reasonable explanation for its extensive involvement as a parent strain in recombination events toward the generation of current prevalent strains in Asia.

## Discussion

To determine the patterns and processes of the evolution of MDV, we presented the complete genomic sequences of 58 MDV strains isolated from 1964 to 2018. Based on the assumption of a co-divergent history with their hosts, the evolutionary rates for herpes simplex virus 1 (HSV-1) have been estimated at 3.5 × 10^–8^ to 3.0 × 10^–9^ subs/site/year (Sakaoka et al., 1994; McGeoch et al., 1995), which is similar to the estimates obtained for the dsDNA of feline papillomaviruses (1.9 × 10^–8^ subs/site/year) (Ong et al., 1993; Rector et al., 2007). In the present study, the mean evolutionary rate of the complete genome of MDV was estimated to be 8.25 × 10^–6^ substitutions per site per year; while Trimpert et al. (2017) determined a mean evolutionary rate of 1.6 × 10^–5^ substitutions per site per year based on the analyses of 22 complete or near- complete MDV genomes. These data indicated that the evolutionary rate of MDV is much higher than that reported for HSV-1 and feline papillomaviruses (Duggan et al., 2016), which might be an important reason for the increased virulence of MDV. It was reported that the evolution of MDV appears to occur periodically in successive waves, resulting in the emergence of more virulent strains capable of breaking through the immune responses induced by the vaccines (Nair, 2005). This finding could help us predict the cycle and approximate time of disease outbreaks through the rate of evolution. According to the regulation of evolution rates and because the evolutionary rates of the clades in 2000 and 2003 were shown to be still at a low level, a major change in MDV virulence could be predicted in the next 10 years or so.

The pattern of the clock-like evolution of MDVs is not unique, but is dominant because most strains have been shown to conform to the model of clock-like evolution. The results of the MCC evolutionary tree showed that the currently prevalent Asian strains had a separate evolutionary path, and based on their derivative status were likely to have evolved from European strains, consistent with the first reported case of MDV in Europe (Biggs, 2001). Moreover, we found that North American and Eurasian strains located in two independent branches with relatively long evolutionary distances, indicating that Eurasian strains had farther evolutionary distances from North American strains and different evolutionary paths. However, we found that a particular strain, ZW2/15, which is known to be currently endemic in Asia, was more closely related to North American strains. This suggested that the strain might have been introduced into Asia from North America, and then continued to spread and evolve in Asia. The European strain ATE2539 located in a separate subclade and not that of the Eurasian clade, which reflected its specificity. When analysing the temporal evolution of each sub-region, the results of the MCC tree showed that the strain distribution based on the UL subregion was most similar to that of the whole genome, followed by the IRS and IRL subregions; whereas the US subregion did not meet the criteria of temporal evolution. This indicated that the evolutionary direction and distribution of MDV strains could be analyzed and predicted using UL subregions instead of the complete genomes. Compared to the UL subregion encoding more than 70 MDV ORFs, the US subregion is shorter that encoding only 11 MDV ORFs including three viral envelope glycoproteins gD, gI, and gE, which might accumulate higher number of mutations than the “housekeeping” genes located in UL and in IR subregions. Moreover, the influence of recombination events on reconstruction of phylogeny from US subregion may be stronger than that for UL due to the much shorter length of US. The MCC tree obtained here revealed the evolutionary history of MDV with high resolution. Such findings have wide-ranging implications, from the successful reconstruction of evolutionary and epidemiological history, to the evolution of virulence.

It is widely known that recombination can lead to the evolution of strains, making them more likely to adapt to new hosts, thus leading to the emergence of new pathogens (Ke et al., 2015). Pairing recombination analyses with an understanding of the epidemiology and pathogenesis of viruses could offer great potential in understanding the importance of recombination and its role in viral evolution. However, some recombination events could still be ignored even when using concatemer analysis. To overcome the problem raised by invisible recombinants, full genome sequencing is deemed necessary (Thiry et al., 2005). In this study, by performing a large-scale complete genome sequence analysis, we found that recombination events were widespread during the evolution of MDVs. We found that many areas of recombination were gradual and not completed in one time; these were observed in some transitional strains. Moreover, some parental strains were shown to be different in their recombination areas, indicating that they were not the direct parent, but had evolved from the parent of the recombination event. As these strains represented a class of similar strains, it was hence assumed that some differential loci existed. In addition, we detected all possible recombination regions and loci, providing accurate regions and information sites for subsequent recombination detection. We found that the genes in the unique region were relatively stable, whereas the breakpoints of the recombination regions located in some highly variable gene coding regions. We considered that their relatively high denaturation was not accidental, but due to recombination. For example, MDV094 (gD), MDV095 (gI), MDV096 (gE), and MDV049 (UL36) genes were all relatively hyper-variant genes with recombination cross sites. Additionally, many recombination occurs in the hinge regions with the repeat fragments, including molecular recombination with retroviral sequences that co-infect the birds (Davidson and Borenshtain, 2001; Kung et al., 2001; Davidson and Silva, 2008). The recombination between MDV and retroviruses including REV and avian leukosis virus is prevalent in China and elsewhere (Davidson et al., 2002; Zhang et al., 2017), which is of importance for their potential to modify the MDV biology and virulence.

Large segment recombination was demonstrated to only occur in the UL and US subregions, with UL conforming to the clock model, whereas US did not. This might have been due to the different effects of recombination, with recombination events being classified with respect to their effect on tree topology, as events that did not change the branch lengths, events that changed the branch lengths but not the tree topology, and events that changed the tree topology (Posada et al., 2002). It was therefore inferred that the recombination events had no effect on the topology of the UL subregion, but had an impact on the US subregion. Although we observed that MDV recombination events were widespread, no recombination was found among the Asiatic clade strains. We assumed that they might have originated from a single strain in a broad sense, and were made prevalent in Asia after recombination with ATE2539 and GA. When a recombination event occurs between two identical strands of DNA, this event is undetectable (Posada et al., 2002). The Eurasian strain ATE2539 was shown to be a result of the recombination of early North American strains and the early Asian strain J-1. Thus, as ATE2539 had the same lineage with Asian strains, this would explain its role as a parent strain in producing the current Asian pandemic strains. Furthermore, ATE2539 was demonstrated to be in an irreplaceable position to participate in the 85743–98986 segment recombination event, further reflecting its uniqueness in the complete genome phylogenetic analysis. In addition, due to the presence of differential loci in the recombination region, ATE2539 was shown to represent a specific type of strain, further highlighting the importance of collecting complete genome sequences. The CVI988 strain also participated in reorganization events as a parent. A Chinese MDV strain derived from the CVI988 vaccine strain through recombination was recently reported (He et al., 2020). These results indicated that recombination events might be an essential evolutionary driving force of MDVs, providing a powerful mechanism for the evolution and adaptation of MDV genomes.

In conclusion, our study identified and characterized the recombination events associated with large-scale complete genomes of MDVs. These results provided evidence of recombination events in the MDV lineage, indicating the importance of recombination for the evolution and genome diversification of MDVs. Our analyses of MDV evolution during a period of 54 years from 1964 to 2018 enabled us to obtain a unique snapshot of the pace of viral evolution in real-time, and our genome scale data provided strong evidence for the rapid evolution of MDVs, highlighting the importance of the continuous surveillance of MDV infections and their evolution.

## Data availability statement

The data presented in this study are deposited in GenBank, and the accession numbers can be found in Supplementary Table 1.

## Author contributions

CL and YZ designed the study. KL and ZY performed the experiments. ZY, KL, YZ, XL, YW, and CL analyzed the data. ZY and KL wrote the manuscript. XW, YG, XQ, HC, and LG participated in discussion of the results. All authors contributed to the article and approved the submitted version.
