# Correction: Complete genome analysis reveals evolutionary history and temporal dynamics of Marek's disease virus

**DOI:** 10.3389/fmicb.2026.1781132

**Published:** 2026-01-20

**Authors:** Kai Li, Zhenghao Yu, Xingge Lan, Yanan Wang, Xiaole Qi, Hongyu Cui, Li Gao, Xiaomei Wang, Yanping Zhang, Yulong Gao, Changjun Liu

**Affiliations:** Avian Immunosuppressive Diseases Division, State Key Laboratory of Veterinary Biotechnology, Harbin Veterinary Research Institute, Chinese Academy of Agricultural Sciences, Harbin, China

**Keywords:** Marek's disease virus, genomic analysis, evolution, recombination, temporal dynamics

An incorrect number was provided for National Natural Science Foundation of China (21A20260). The correct number is (U21A20260).

The original version of this article has been updated.

