# Supplementary material for: Complete genome analysis reveals evolutionary history and temporal dynamics of Marek’s disease virus
Source: Front Microbiol. 2022 Nov 3;13:1046832. doi: 10.3389/fmicb.2022.1046832 (PMC9669313; doi:10.3389/fmicb.2022.1046832)
Supplement: Supplementary file 2 [file Table_2.docx]

**Table S2**. The evolutionary rate of MDV complete genomes analyzed with a GTR+Γ4 site model.

|  | Mean Rate | 95% HPD (-) | 95% HPD (+) | 95% HPD (-) | 95% HPD (+) |
| --- | --- | --- | --- | --- | --- |
| HYK+Γ4 | 7.71×10^-6^ | 6.24×10^-6^ | 9.16×10^-6^ | 1.48×10^-6^ | 1.45×10^-6^ |
| HYK+Γ4 | 7.67×10^-6^ | 6.11×10^-6^ | 9.15×10^-6^ | 1.57×10^-6^ | 1.47×10^-6^ |
| GTR+Γ4 | 7.68×10^-6^ | 6.19×10^-6^ | 9.16×10^-6^ | 1.49×10^-6^ | 1.48×10^-6^ |
| GTR+Γ4 | 7.62×10^-6^ | 6.19×10^-6^ | 9.17×10^-6^ | 1.43×10^-6^ | 1.55×10^-6^ |
| HYK+Γ4 | 9.52×10^-6^ | 3.94×10^-6^ | 1.93×10^-5^ | 5.58×10^-6^ | 9.74×10^-6^ |
| HYK+Γ4 | 8.71×10^-6^ | 2.22×10^-6^ | 2.35×10^-5^ | 6.49×10^-6^ | 1.47×10^-5^ |
| GTR+Γ4 | 8.25×10^-6^ | 3.78×10^-6^ | 1.56×10^-5^ | 4.47×10^-6^ | 7.39×10^-6^ |
| GTR+Γ4 | 8.43×10^-6^ | 4.57×10^-6^ | 1.30×10^-5^ | 3.87×10^-6^ | 4.61×10^-6^ |
