# Supplementary material for: Complete genome analysis reveals evolutionary history and temporal dynamics of Marek’s disease virus
Source: Front Microbiol. 2022 Nov 3;13:1046832. doi: 10.3389/fmicb.2022.1046832 (PMC9669313; doi:10.3389/fmicb.2022.1046832)
Supplement: Supplementary file 3 [file Presentation_1.pptx]

## Slide 1
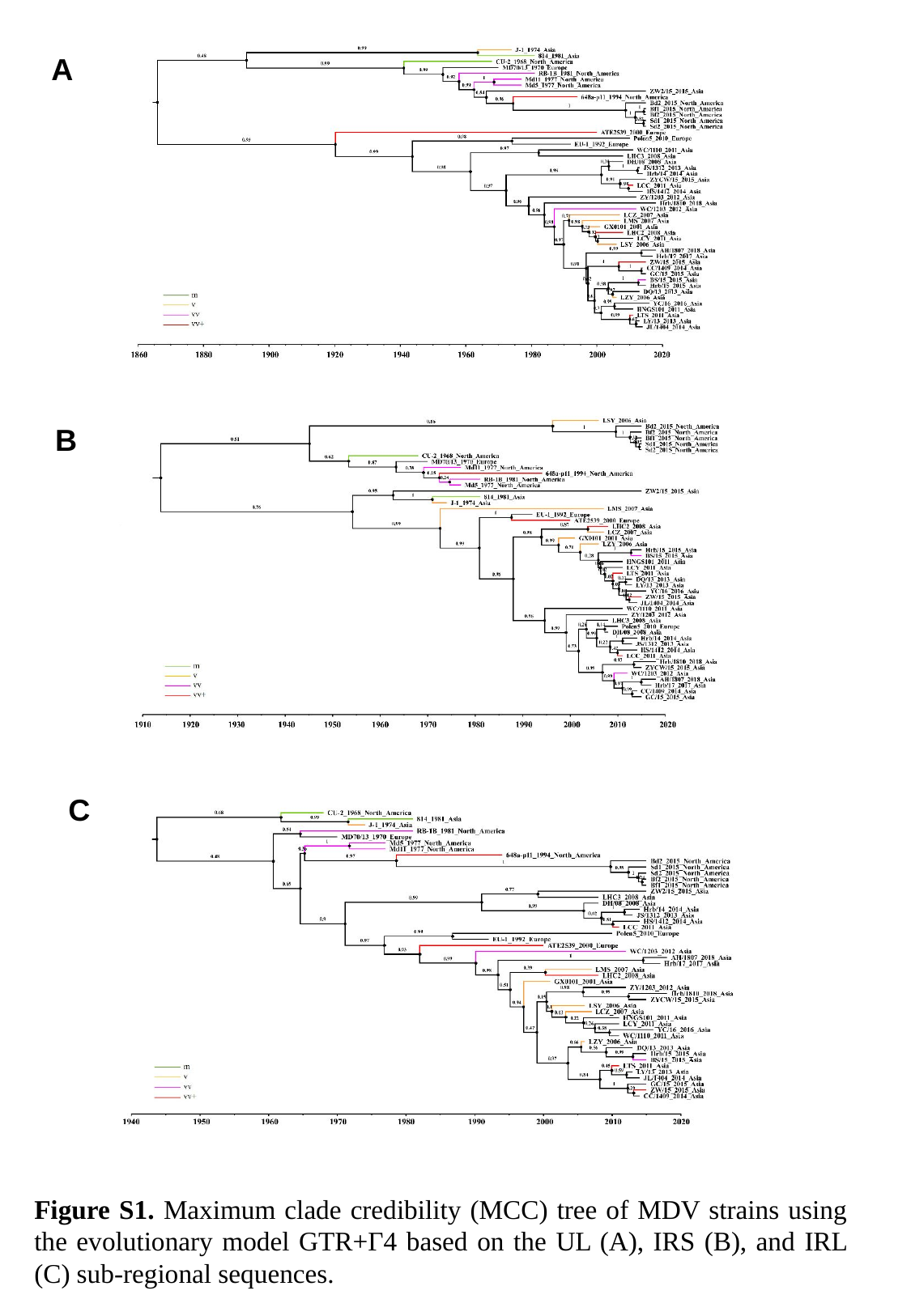

A
B
C
Figure S1. Maximum clade credibility (MCC) tree of MDV strains using the evolutionary model GTR+Γ4 based on the UL (A), IRS (B), and IRL (C) sub-regional sequences.
