# Supplementary figures and images for: Complete genome analysis reveals evolutionary history and temporal dynamics of Marek’s disease virus
Source: Front Microbiol. 2022 Nov 3;13:1046832. doi: 10.3389/fmicb.2022.1046832 (PMC9669313; doi:10.3389/fmicb.2022.1046832)

## Slide 1
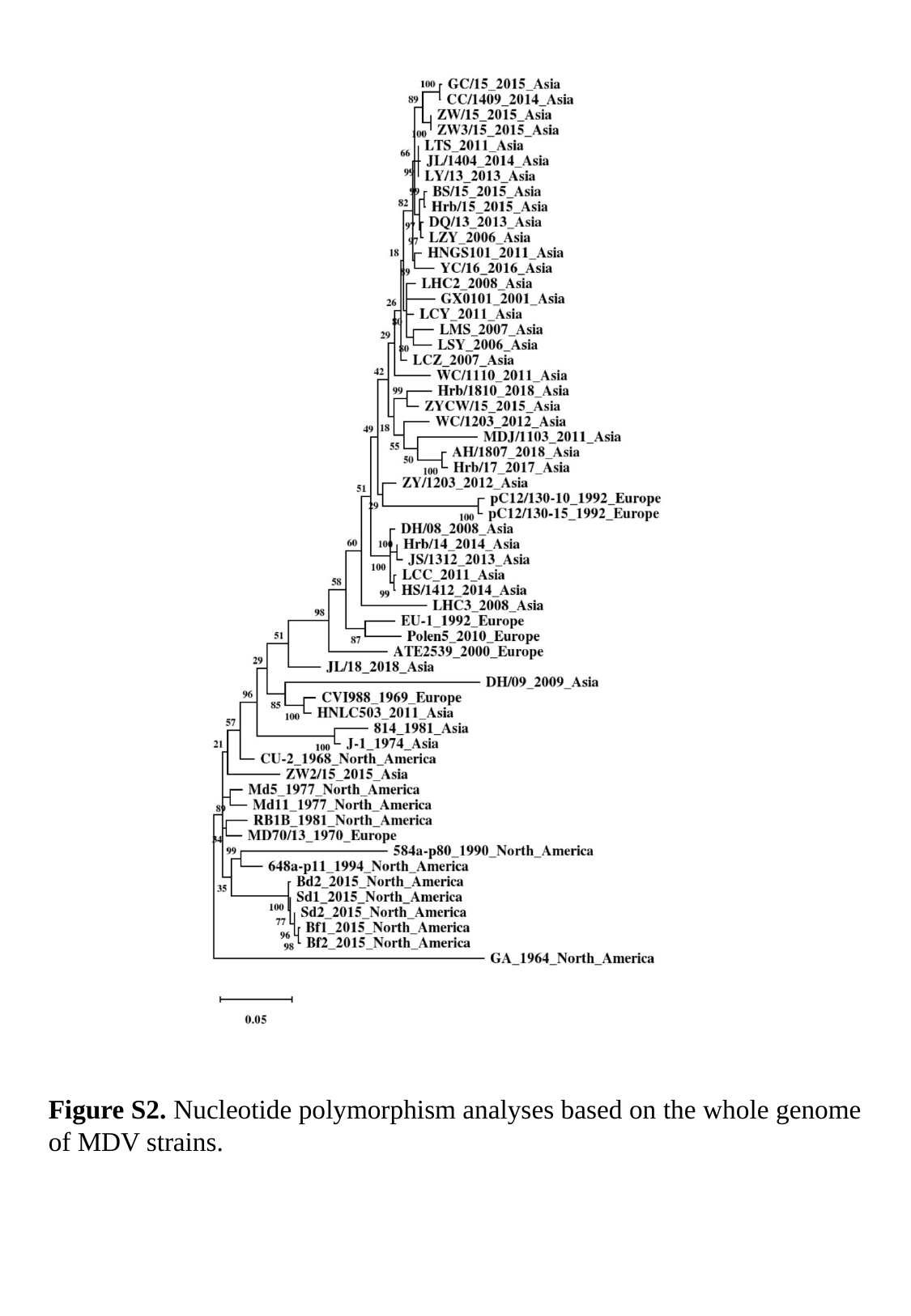

Figure S2. Nucleotide polymorphism analyses based on the whole genome of MDV strains.

Supplement: Supplementary file 4 [file Presentation_2.PPTX]

## Slide 1
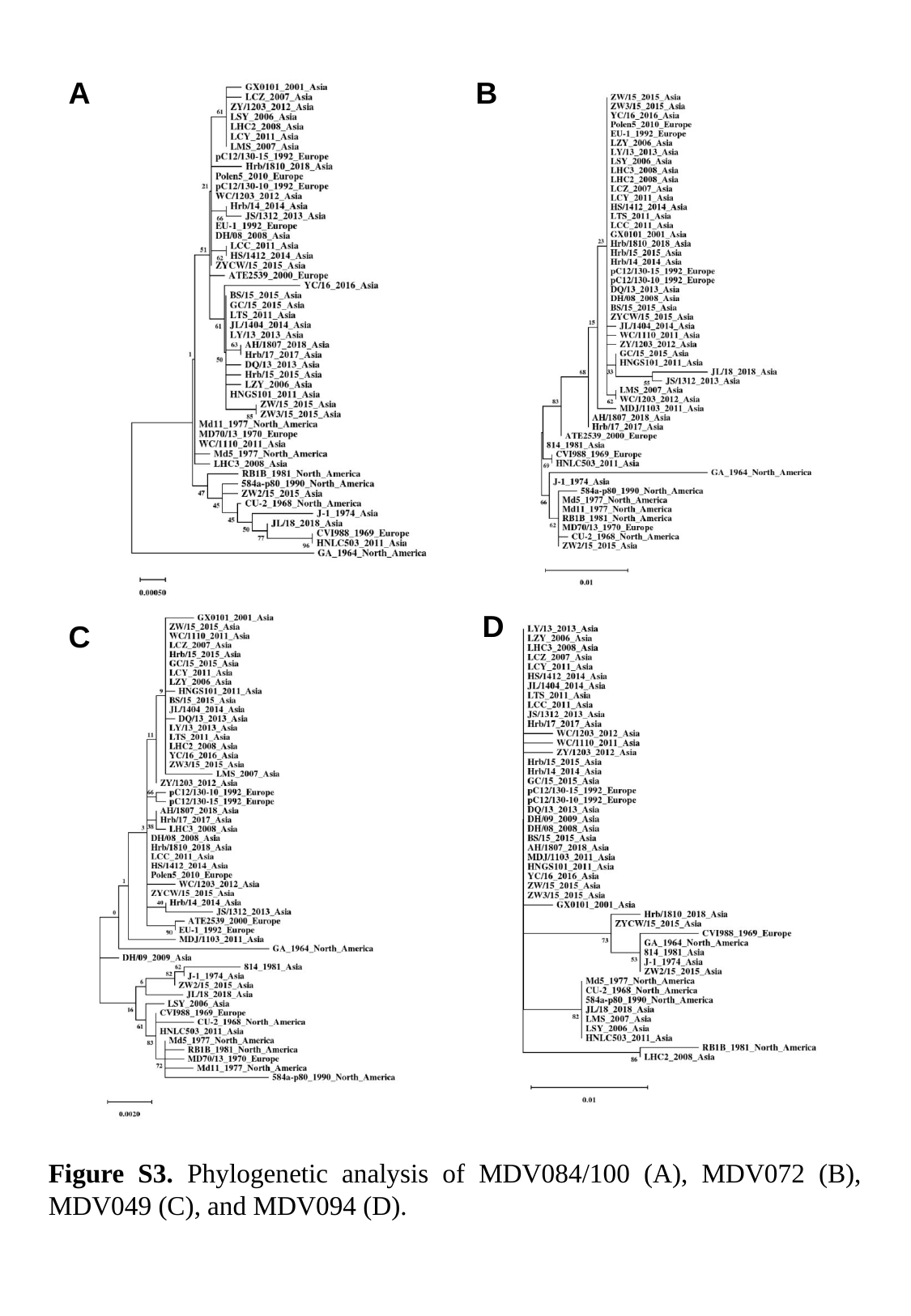

A
B
D
C
Figure S3. Phylogenetic analysis of MDV084/100 (A), MDV072 (B), MDV049 (C), and MDV094 (D).

Supplement: Supplementary file 5 [file Presentation_3.PPTX]
