# Supplementary material for: Complete genome analysis reveals evolutionary history and temporal dynamics of Marek’s disease virus
Source: Front Microbiol. 2022 Nov 3;13:1046832. doi: 10.3389/fmicb.2022.1046832 (PMC9669313; doi:10.3389/fmicb.2022.1046832)
Supplement: Supplementary file 6 [file Presentation_4.pptx]

## Slide 1
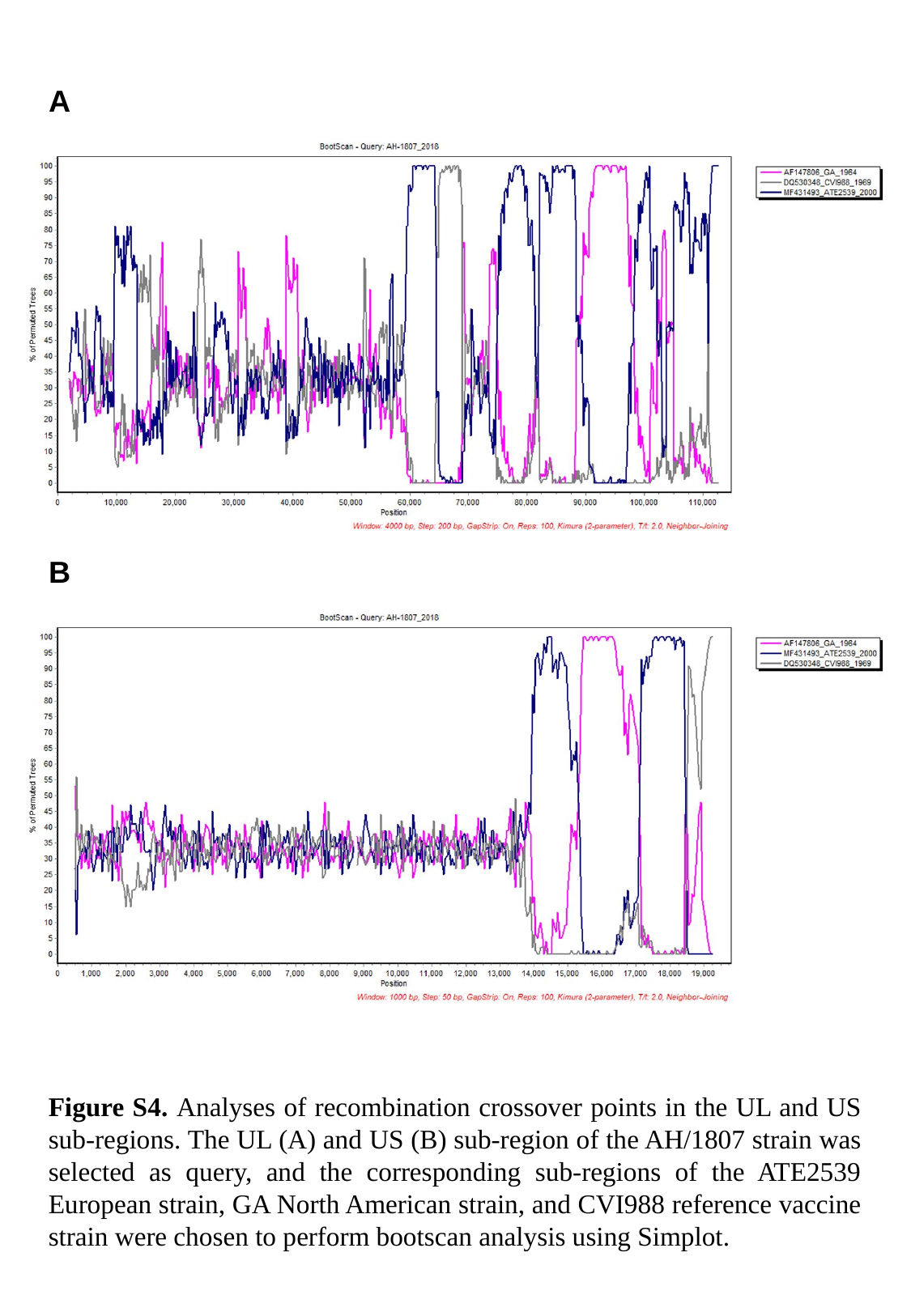

A
B
Figure S4. Analyses of recombination crossover points in the UL and US sub-regions. The UL (A) and US (B) sub-region of the AH/1807 strain was selected as query, and the corresponding sub-regions of the ATE2539 European strain, GA North American strain, and CVI988 reference vaccine strain were chosen to perform bootscan analysis using Simplot.
