# Supplementary material for: Complete genome analysis reveals evolutionary history and temporal dynamics of Marek’s disease virus
Source: Front Microbiol. 2022 Nov 3;13:1046832. doi: 10.3389/fmicb.2022.1046832 (PMC9669313; doi:10.3389/fmicb.2022.1046832)
Supplement: Supplementary file 7 [file Presentation_5.PPTX]

## Slide 1
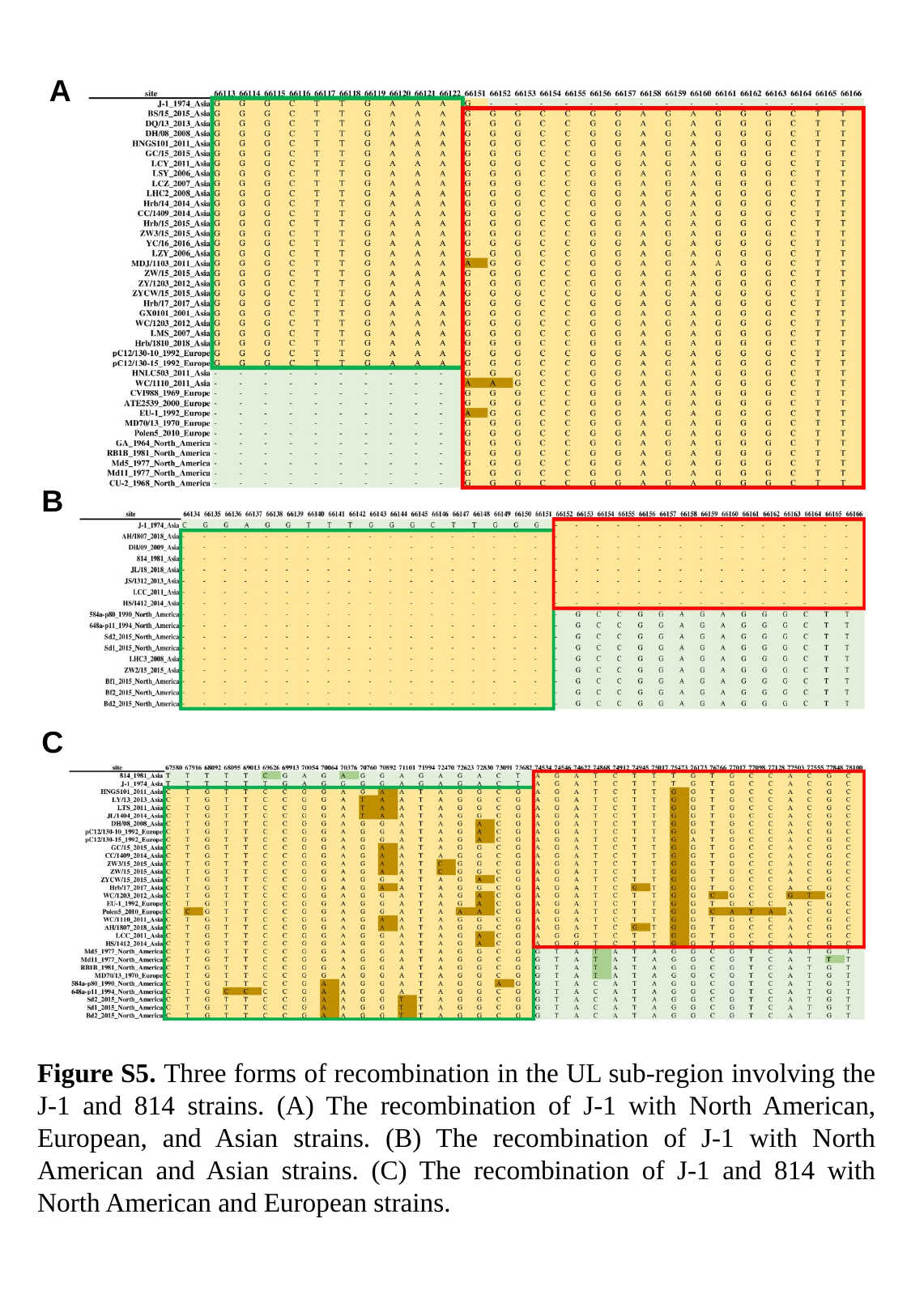

A
B
C
Figure S5. Three forms of recombination in the UL sub-region involving the J-1 and 814 strains. (A) The recombination of J-1 with North American, European, and Asian strains. (B) The recombination of J-1 with North American and Asian strains. (C) The recombination of J-1 and 814 with North American and European strains.
