# Supplementary material for: Complete genome analysis reveals evolutionary history and temporal dynamics of Marek’s disease virus
Source: Front Microbiol. 2022 Nov 3;13:1046832. doi: 10.3389/fmicb.2022.1046832 (PMC9669313; doi:10.3389/fmicb.2022.1046832)
Supplement: Supplementary file 8 [file Presentation_6.PPTX]

## Slide 1
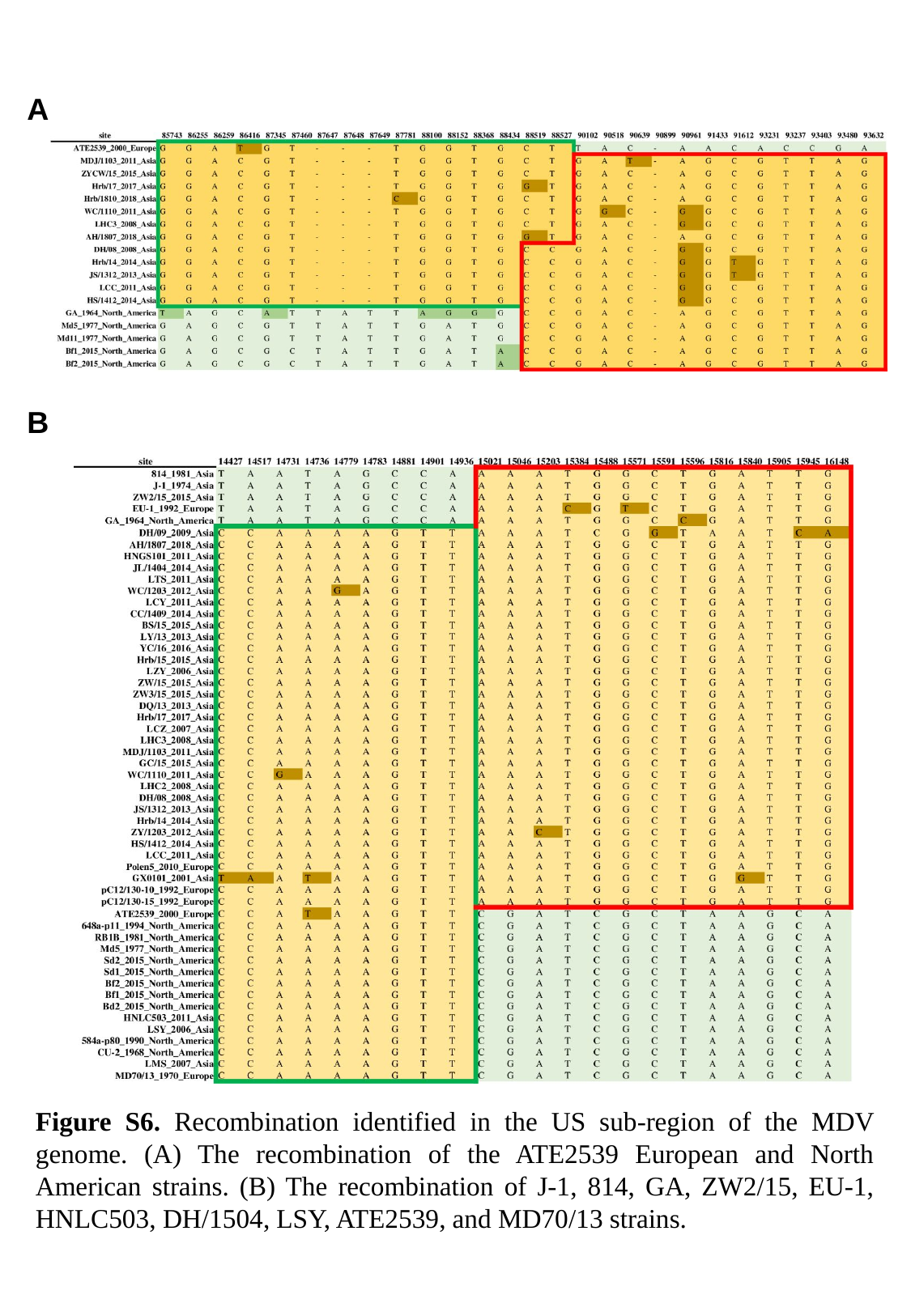

A
B
Figure S6. Recombination identified in the US sub-region of the MDV genome. (A) The recombination of the ATE2539 European and North American strains. (B) The recombination of J-1, 814, GA, ZW2/15, EU-1, HNLC503, DH/1504, LSY, ATE2539, and MD70/13 strains.
