# Supplementary material for: Complete genome analysis reveals evolutionary history and temporal dynamics of Marek’s disease virus
Source: Front Microbiol. 2022 Nov 3;13:1046832. doi: 10.3389/fmicb.2022.1046832 (PMC9669313; doi:10.3389/fmicb.2022.1046832)
Supplement: Supplementary file 9 [file Presentation_7.PPTX]

## Slide 1
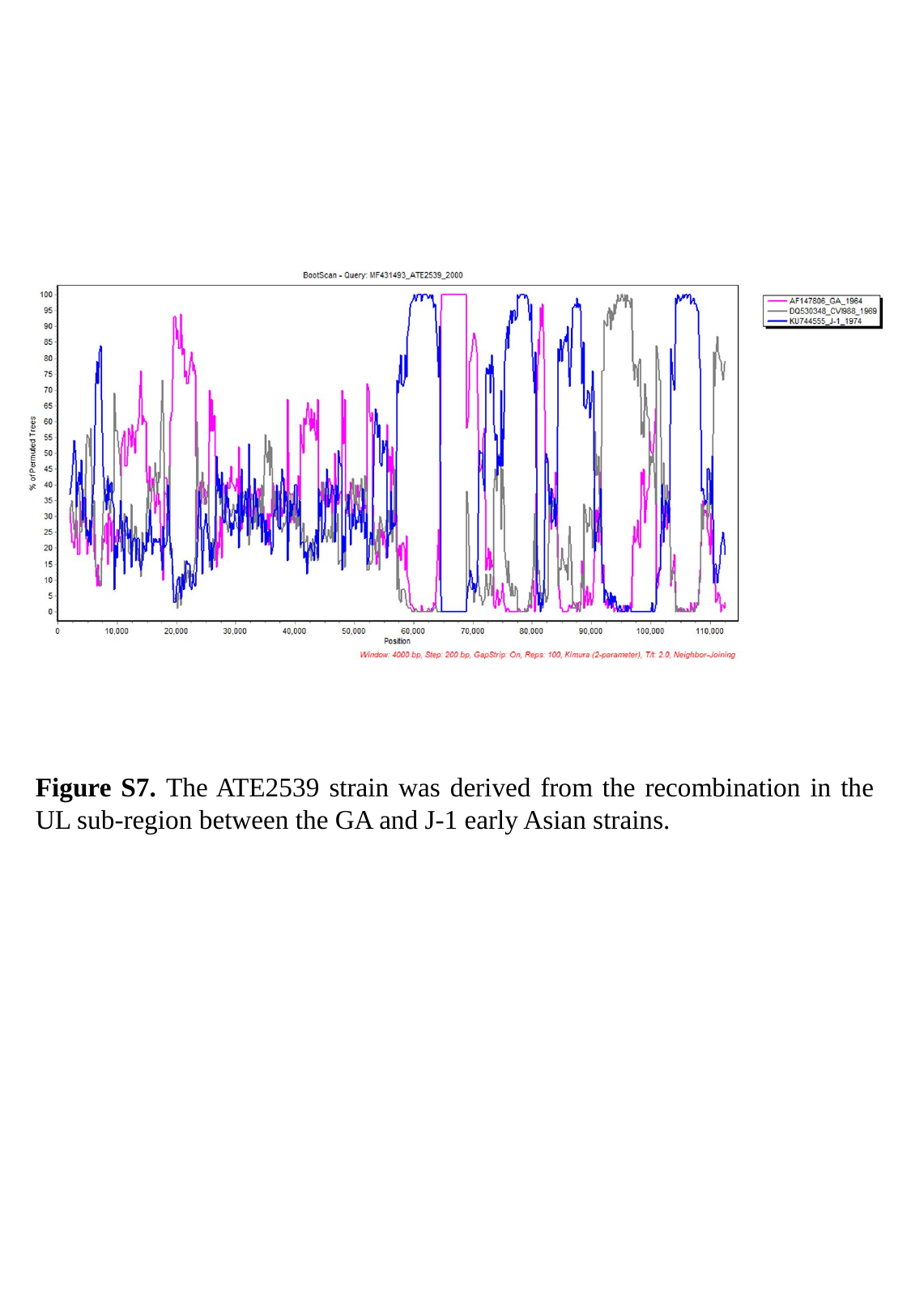

Figure S7. The ATE2539 strain was derived from the recombination in the UL sub-region between the GA and J-1 early Asian strains.
